# Sodium Meta-Arsenite Ameliorates Hyperglycemia in Obese Diabetic *db/db* Mice by Inhibition of Hepatic Gluconeogenesis

**DOI:** 10.1155/2014/961732

**Published:** 2014-12-24

**Authors:** Young-Sun Lee, Eun-Kyu Lee, Hyun-Hee Oh, Cheol Soo Choi, Sujong Kim, Hee-Sook Jun

**Affiliations:** ^1^Lee Gil Ya Cancer and Diabetes Institute, Gachon University, 7-45 Songdo-dong, Yeonsu-gu, Incheon 406-840, Republic of Korea; ^2^Endocrinology, Internal Medicine, Gachon University Gil Medical Center, Namdong-gu, Guwol-dong 1198, Incheon 405-760, Republic of Korea; ^3^Gachon Medical Research Institute, Gil Hospital, Incheon 405-760, Republic of Korea; ^4^Komipharm International Co. Ltd., 3188 Seongnam-dong, Jungwon-gu, Seongnam-si, Gyeonggi-do 462-827, Republic of Korea; ^5^College of Pharmacy and Gachon Institute of Pharmaceutical Science, Gachon University, 7-45 Songdo-dong, Yeonsu-gu, Incheon 406-840, Republic of Korea

## Abstract

Sodium meta-arsenite (SA) is implicated in the regulation of hepatic gluconeogenesis-related genes *in vitro*; however, the effects *in vivo* have not been studied. We investigated whether SA has antidiabetic effects in a type 2 diabetic mouse model. Diabetic *db/db* mice were orally intubated with SA (10 mg kg^−1^ body weight/day) for 8 weeks. We examined hemoglobin A1c (HbA1c), blood glucose levels, food intake, and body weight. We performed glucose, insulin, and pyruvate tolerance tests and analyzed glucose production and the expression of gluconeogenesis-related genes in hepatocytes. We analyzed energy metabolism using a comprehensive animal metabolic monitoring system. SA-treated diabetic *db/db* mice had reduced concentrations of HbA1c and blood glucose levels. Exogenous glucose was quickly cleared in glucose tolerance tests. The mRNA expressions of genes for gluconeogenesis-related enzymes, glucose 6-phosphatase (G6Pase), and phosphoenolpyruvate carboxykinase (PEPCK) were significantly reduced in the liver of SA-treated diabetic *db/db* mice. In primary hepatocytes, SA treatment decreased glucose production and the expression of G6Pase, PEPCK, and hepatocyte nuclear factor 4 alpha (HNF-4*α*) mRNA. Small heterodimer partner (SHP) mRNA expression was increased in hepatocytes dependent upon the SA concentration. The expression of Sirt1 mRNA and protein was reduced, and acetylated forkhead box protein O1 (FoxO1) was induced by SA treatment in hepatocytes. In addition, SA-treated diabetic *db/db* mice showed reduced energy expenditure. Oral intubation of SA ameliorates hyperglycemia in *db/db* mice by reducing hepatic gluconeogenesis through the decrease of Sirt1 expression and increase in acetylated FoxO1.

## 1. Introduction

Diabetes mellitus is a metabolic disease characterized by hyperglycemia, which results from defects in insulin secretion from pancreatic beta cells, insulin resistance in peripheral tissues, and increased glucose production by the liver [1–3]. The liver plays a critical role in the maintenance of glucose homeostasis by balancing the uptake, storage, and release of glucose [4]. Fasting or starvation induces glucose synthesis in the liver through glycogenolysis and gluconeogenesis [5]. However, elevated hepatic glucose production is associated with the pathogenesis of type 2 diabetes [6, 7]. In this process, glucose-6-phosphatase (G6Pase) catalyzes the terminal step in the glycogenolytic and gluconeogenic pathways, and phosphoenolpyruvate carboxykinase (PEPCK) is a key regulatory enzyme driving gluconeogenesis [8, 9]. Insulin suppresses gluconeogenesis by inhibiting the transcription of PEPCK and G6Pase [10, 11].

Arsenic trioxide has a long history as biomedical interest and is approved by the Food and Drug Administration (FDA) for treatment of certain leukemias [12, 13]. Sodium meta-arsenite (NaAsO_2_) is produced by dissolving arsenic trioxide. Sodium meta-arsenite (SA, KML001) has entered phase II clinical trials for the treatment of solid tumors and hematopoietic malignancies. In addition, sodium meta-arsenite (SA) is reported to have insulin-mimetic effects on glucose homeostasis. SA inhibits forskolin/dexamethasone-induced PEPCK and G6Pase gene expression in hepatic cell lines and rat primary hepatocytes [14, 15]. SA activates AMP-activated protein kinase [15, 16], which in turn induces small heterodimer partner (SHP) which inhibits the expression of hepatic gluconeogenic genes, and this repression is abolished by SHP inhibition [15]. SA also suppresses dexamethasone-induced PEPCK transcription in 14-day chick embryo livers* in vivo* [17].

Despite the demonstrated effects of SA in reducing the expression of gluconeogenesis genes, the antidiabetic effect of SA in type 2 diabetes has not yet been evaluated* in vivo*. In this study, we examined the therapeutic effect of SA in diabetic* db/db* mice, an animal model of human type 2 diabetes, as well as the mechanisms involved in the improvement of hepatic gluconeogenesis.

## 2. Materials and Methods

### 2.1. Animals

*db/db* mice were obtained from the Korea Research Institute of Bioscience and Biotechnology (Daejeon, Korea) and* C57BL/6 *mice were obtained from the Orient Bio Inc. (Gyeonggi, Korea) and maintained in specific pathogen-free conditions at the animal facility at Gachon University of Medicine and Science under a 12 h light : 12 h dark photoperiod. Animals were fed* ad libitum* on a standard rodent diet. The* db/db* male mice (aged 6–8 weeks) were monitored for the development of hyperglycemia using a glucometer (One Touch Ultra; LifeScan Inc., Milpitas, CA, USA). Pair-fed diabetic* db/db* mice were given the same daily amount of food as that eaten by the corresponding SA-treated group during the previous day. All animal experiments were carried out under a protocol approved by the Institutional Animal Care and Use Committee at the Gachon University of Medicine and Science. A total of 68 animals were used in the experiments described here.

### 2.2. Treatment with SA

Six- to eight-week-old diabetic male* db/db* mice (random blood glucose levels > 300 mg dL^−1^ for 3 consecutive days) were orally intubated with SA (10 mg kg^−1^ body weight/day; Komipharm, Seoul, Korea) or phosphate buffered saline (PBS) for 8 weeks. Food consumption was measured weekly. After 4 and 8 weeks of treatment, glucose levels were measured following the removal of food for 14 h. All animal groups were body weight-matched with the SA-treated group at the beginning of each experiment.

### 2.3. Blood Analysis

After 8 weeks of SA treatment, mice were not fed for 4 h, and blood samples were drawn into heparinized capillary tubes from the periorbital veins. The whole blood was used for HbA1c measurements, and serum was used for alanine aminotransferase (ALT) and aspartate aminotransferase (AST) measurements using the AU480 Chemistry System (Beckman Coulter Life Sciences, California, USA).

### 2.4. Glucose, Insulin, and Pyruvate Tolerance Tests

After 4 and 8 weeks of SA treatment, mice were not fed for 14 h, and then a glucose solution (2 g kg^−1^ body weight) was injected intraperitoneally. Blood glucose levels were measured at 0, 30, 60, 90, 150, and 180 min after glucose injection at 9:00 a.m. For insulin tolerance tests, mice were not fed for 4 h and were injected with insulin (2 units kg^−1^ body weight, i.p.), and blood glucose levels were measured at 0, 30, 60, and 90 min after insulin injection at 1:00 p.m. For pyruvate tolerance tests, C57BL/6 mice were orally intubated with SA (10 mg kg^−1^ body weight) or PBS and then fasted overnight. Fifteen hours after oral intubation, mice were injected i.p. with 1 g kg^−1^ body weight of sodium pyruvate in PBS, and blood glucose levels were measured at 0, 15, 30, 60, 90, and 120 min after pyruvate injection at 9:00 a.m.

### 2.5. Real-Time Quantitative PCR (RT-qPCR)

Total RNA was isolated from the liver of SA-treated mice or hepatocytes from C57BL/6 mice, and cDNA was synthesized using the PrimeScript First-Strand cDNA Synthesis Kit (TaKaRa Bio, Inc., Otsu, Japan). PCR was carried out in a 7900HT fast real-time PCR system (Applied Biosystems, Carlsbad, CA) at 95°C for 10 min, followed by 40 cycles at 95°C for 15 s, 60°C for 1 min. As an internal control, cyclophilin mRNA was amplified. The specific PCR primers were G6Pase: sense 5′-GTGTTGACATCGGCCC-3′, antisense 5′-AACTGAAGCCGGTTAG-3′; PEPCK: sense 5′-CGCAAGCTGAAGAAATATGACAA3′, antisense 5′-TCGATCCTGGCCACATCTC-3′; hepatocyte nuclear factor-4*α* (HNF-4*α*): sense 5′-CCAACCTCAATTCATCCAACA-3′, antisense 5′-CCCGGTCCGCCACAGAT-3′; SHP: sense 5′-AGG AACCTGCCGTTCCTTCTG-3, antisense 5′-TGG CTT CCT CTA GCA GGA TC-3′; Sirt1: sense 5′-TTGGTGGTACAAACAGGTATTGA-3′, antisense 5′-CAGTGAGAAAATGCTGGCCTA-3′; AgRP: sense 5′-TGCTACTGCCGCTTCTTCAA-3′, antisense 5′-CTTTGCCCAAACAACATCCA-3′; POMC: sense 5′-GAGGCCACTGAACATCTTTGTC-3′, antisense 5′-GCAGAGGCAAACAAGATTGG; MC4R: sense 5′-TGCTGGTGAGCGTTTCGA-3′, antisense 5′-GGCATCCGTATCCGTACT-3′; and cyclophilin: sense 5′-TGGAGAGCACCAAGACAGACA-3′, antisense 5′-TGCCGGAGTCGACAATGAT-3′. The relative copy number was calculated using the threshold crossing point (Ct) as calculated by the 7900HT fast real-time PCR software combined with the delta delta Ct calculations.

### 2.6. Glucose Production Assay

Primary hepatocytes were isolated from C57BL/6 mice by collagenase perfusion. For the hepatic glucose production assay, hepatocytes were seeded in 6-well plates at a density of 2 × 10^5^ cells 2 mL^−1^/well and cultured in Hepatozyme SFM media (Gibco) for 24 h; unattached cells were discarded. Hepatocytes were then treated for 24 h with SA (5 or 10 *μ*M). After 24 h of culture, cells were washed with PBS and then cultured in glucose-free DMEM supplemented with 20 mM sodium lactate and 2 mM sodium pyruvate for 2 h. Glucose concentration in the media was measured by a glucose assay kit (BioVision Research Products, Mountain View, CA).

### 2.7. Western Blot Analysis

Hepatocyte cell lysates were preparedand subjected to sodium dodecyl sulfate-polyacrylamide gel electrophoresis. Proteins were electrotransferred onto nitrocellulose membranes. Membranes were incubated with anti-Sirt1, anti-acetyl-FoxO1, or anti-FoxO1 antibodies (Santa Cruz Biotechnology, Santa Cruz, CA) overnight at 4°C. After washing, membranes were incubated with a horseradish peroxidase-conjugated secondary antibody (anti-rabbit IgG, Chemicon International, Temecula, CA) for 1 h at room temperature. Reactive bands were detected by enhanced chemiluminescence (Thermo Fisher Scientific, Rockford, IL).

### 2.8. Indirect Calorimetry

A comprehensive animal metabolic monitoring system (CLAMS; Columbus Instruments, Columbus, OH) was used to evaluate energy expenditure, respiratory exchange ratio, and locomotor activity. Energy expenditure was measured by assessing oxygen consumption with indirect calorimetry. The respiratory exchange ratio was computed as carbon dioxide output (VCO_2_) divided by oxygen consumption (VO_2_). Locomotor activity was measured on *x*- and *z*-axis by using infrared beams to count the beam breaks during 72 h.

### 2.9. Cytotoxicity and Proliferation Assays

For cytotoxicity and proliferation assays, hepatocytes were seeded in 96-well plates at a density of 2 × 10^4^ cells 100 *μ*L^−1^/well and incubated for 24 h. The cells were then incubated in culture medium containing SA (5 or 10 *μ*M). Following 24 h of incubation, cell viability was determined using a Cell Counting Kit-8 (Dojindo Laboratories, Kumamoto, Japan) according to the manufacturer's protocol. For the ^3^H-thymidine incorporation assay, hepatocytes were seeded and incubated as described above with the addition of ^3^H-thymidine (1 *μ*Ci/well). After 24 h of incubation, the cells were washed with PBS and then analyzed for ^3^H-thymidine incorporation using a scintillation beta-counter, 1450 LSC and Luminescence Counter MicroBeta TriLux (Perkin Elmer).

### 2.10. Glucose-Stimulated Insulin Secretion

After 8 weeks of SA treatment, mice were not fed for 14 h, and then a glucose solution (2 g kg^−1^ body weight) was injected intraperitoneally. Blood glucose levels were measured at 0 and 30 min after glucose injection. The concentration of serum insulin was measured by an ultrasensitive mouse insulin enzyme immunosorbent assay kit (ALPCO, Windham, NH).

### 2.11. Statistical Analysis

Data are presented as mean ± SE. Statistical significance of the difference between two groups was analyzed by unpaired Student's *t*-test for comparison of two groups or ANOVA followed by Fisher's protected least significant difference test for multiple groups. *P* < 0.05 was accepted as significant.

## 3. Results

### 3.1. Reduction of Blood Glucose Level and Food Intake in SA-Treated* db/db* Mice

**Figure 1:**
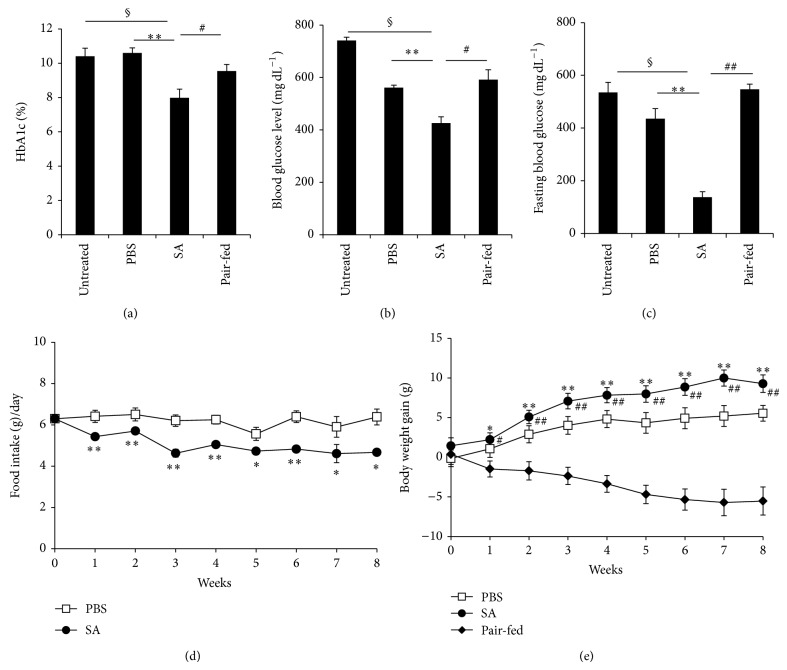
Decrease of blood glucose levels and food intake in SA-treated* db/db* mice. Diabetic* db/db* mice were orally intubated with SA (10 mg kg^−1^ body weight/day) or PBS for 8 weeks. (a) Blood levels of HbA1c, (b) nonfasting blood glucose levels, (c) fasting blood glucose levels, (d) food intake, and (e) body weights were measured. Untreated diabetic* db/db* mice and pair-fed diabetic* db/db* mice served as controls. *n* = 5–11 per group. ^*^ *P* < 0.05, ^**^ *P* < 0.01 compared with PBS-treated mice; ^#^ *P* < 0.05, ^##^ *P* < 0.01 compared with the pair-fed group; ^§^ *P* < 0.01, compared with untreated mice.

To investigate the effects of SA on a type 2 diabetic mouse model, we orally intubated diabetic* db/db* mice with SA daily and measured HbA1c levels, glucose levels, food intake, and body weight. We found that HbA1c levels of SA-treated mice were significantly lower compared with the untreated, PBS-treated, and pair-fed control groups at 8 weeks of treatment (Figure 1(a)). Blood glucose levels were unchanged among groups at 4 weeks of treatment but were significantly lower in the SA-treated group at 8 weeks, compared with the control groups (Figure 1(b)). Fasting blood glucose levels were also significantly decreased in SA-treated mice compared with untreated, PBS-treated mice, and pair-fed mice at 8 weeks of treatment (Figure 1(c)). Interestingly, food intake in SA-treated mice was significantly decreased compared with PBS-treated mice (Figure 1(d)), but body weight gain was significantly increased at 8 weeks of SA treatment (Figure 1(e)). In contrast, the pair-fed group had significantly lower body weights as compared with the PBS-treated group (Figure 1(e)). In addition, the blood concentrations of ALT and AST, indicators of liver damage, were not different between SA- and PBS-treated* db/db* mice (see Supplementary Figure 1  in Supplementary Material available online at http://dx.doi.org/10.1155/2014/961732). These results suggest that treatment with 10 mg kg^−1^ of SA for 8 weeks may not have toxic effects in* db/db* mice. In low concentrations, SA has been reported to cause transient stimulation of cell growth [18]. In our study, we also found that SA in low doses (5 *μ*M) stimulated hepatocyte cell growth and did not show cell toxicity, even at 10 *μ*M (Supplementary Figure 2).

### 3.2. Improvement of Glucose Tolerance in SA-Treated* db/db* Mice

**Figure 2:**
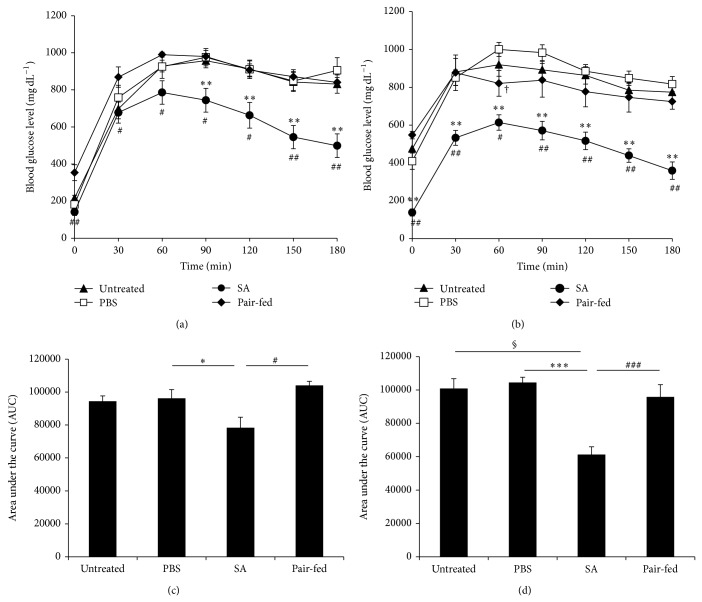
SA improved glucose tolerance in diabetic* db/db* mice. Diabetic* db/db* mice were orally intubated with SA (10 mg kg^−1^ body weight/day) or PBS. (a) Four weeks and (b) 8 weeks later, mice were not fed for 4 h and were injected with glucose, and blood glucose levels were measured (*n* = 5–10 per group). The area under the curve measured at (c) 4 weeks and (d) 8 weeks. Untreated diabetic* db/db* mice and pair-fed diabetic* db/db* mice served as controls. ^*^ *P* < 0.05, ^**^ *P* < 0.01, and ^***^ *P* < 0.0001 compared with PBS-treated mice; ^#^ *P* < 0.05, ^##^ *P* < 0.01, and ^###^ *P* < 0.005 compared with the pair-fed group;  ^†^ *P* < 0.05 pair-fed mice compared with PBS-treated mice; ^§^ *P* < 0.01, compared with untreated mice.

To determine whether blood glucose levels are properly controlled in SA-treated* db/db* mice, we performed intraperitoneal glucose tolerance tests at 4 and 8 weeks of SA treatment. Blood glucose levels in SA-treated mice were significantly lower at all time points following glucose injection compared with pair-fed mice and significantly lower after 90 min compared with PBS-treated mice at 4 weeks (Figure 2(a)). At 8 weeks of treatment, blood glucose levels of SA-treated mice were significantly reduced at all time points compared with PBS-treated and pair-fed mice (Figure 2(b)). Blood glucose levels in the pair-fed group were not significantly different from the untreated or PBS-treated groups at any time point, except at 60 min after glucose loading (Figure 2(b)). The area under the curve was significantly reduced in SA-treated mice compared with PBS-treated and pair-fed mice at both 4 and 8 weeks of treatment (Figures 2(c) and 2(d)).

**Figure 3:**
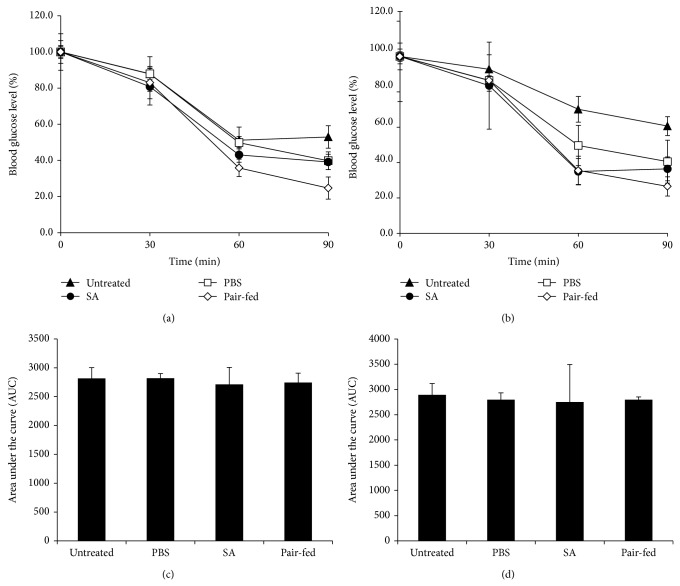
SA treatment did not improve insulin sensitivity in diabetic* db/db* mice. Diabetic* db/db* mice were orally intubated with SA (10 mg kg^−1^ body weight/day) or PBS. (a) Four weeks and (b) 8 weeks later, insulin was injected and blood glucose levels were measured (*n* = 4–10 per group). Data are expressed as a percentage of the initial blood glucose level before insulin injection. The area under the curve measured at (c) 4 weeks and (d) 8 weeks. Untreated diabetic* db/db* mice and pair-fed diabetic* db/db* mice served as controls.

To address whether SA treatment improves insulin sensitivity, we performed insulin tolerance tests at 4 and 8 weeks of SA treatment. Glucose reduction in SA-treated mice was not different from untreated, PBS-treated, or pair-fed mice at 4 weeks or 8 weeks of treatment (Figures 3(a) and 3(b)). Similarly, the area under the curves was not different among groups (Figures 3(c) and 3(d)). To measure insulin secretion in SA-treated mice, we analyzed glucose-stimulated insulin secretion after 8 weeks of SA treatment. Insulin secretion was not different between the SA- and PBS-treated groups (Supplementary Figure 3).

### 3.3. Decreased Expression of Gluconeogenic Genes in SA-Treated* db/db* Mice and Reduction of Glucose Production and Expression of Gluconeogenesis-Related Genes in SA-Treated Hepatocytes

**Figure 4:**
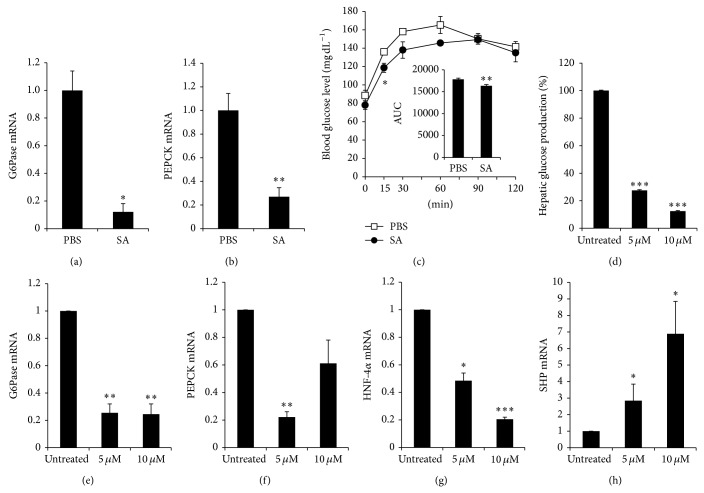
SA suppressed gluconeogenic gene expression in the liver of SA-treated* db/db* mice and inhibited glucose production and gluconeogenic gene expression in hepatocytes. Diabetic* db/db* mice were orally intubated with SA (10 mg kg^−1^ body weight/day) or PBS. Eight weeks later, the liver tissue was removed. The mRNA expression of (a) G6Pase and (b) PEPCK was analyzed by RT-qPCR and normalized by cyclophilin expression. The fold change was calculated as a ratio of the expression level in PBS-treated diabetic* db/db* mice. ^*^ *P* < 0.005, ^**^ *P* < 0.0001 compared with PBS-treated group (*n* = 4–9/group). (c) For pyruvate tolerance tests, C57BL/6 mice were orally intubated with SA (10 mg kg^−1^ body weight) or PBS and then fasted overnight. Fifteen hours after SA oral intubation, mice were injected i.p. with 1 g kg^−1^ body weight of sodium pyruvate in PBS, and blood glucose levels were measured at 0, 15, 30, 60, 90, and 120 min after pyruvate injection (*n* = 3-4 per group). The area under the curve (AUC) was calculated. ^*^ *P* < 0.05 and ^**^ *P* < 0.01, compared with PBS-treated mice. (d) Primary hepatocytes from C57BL/6 mice were incubated with 5 or 10 *μ*M SA for 48 h and cultured in glucose-free DMEM supplemented with 20 mM sodium lactate and 2 mM sodium pyruvate for 2 h. Glucose production in media was measured and normalized by the amount of total protein. Relative glucose production was calculated as a percent of glucose production from untreated hepatocytes. Primary hepatocytes from C57BL/6 mice were incubated with 5 or 10 *μ*M SA for 24 h, and the expression of (e) G6Pase, (f) PEPCK, (g) HNF-4*α*, and (h) SHP mRNA was analyzed by RT-qPCR, with values normalized to cyclophilin expression. The fold change was calculated as ratio of the expression in untreated hepatocytes. Data are mean ± SE from three to four independent experiments. ^*^ *P* < 0.05, ^**^ *P* < 0.01, and ^***^ *P* < 0.001 compared with untreated hepatocytes.

To determine whether SA treatment affects the expression of genes involved in glucose production, we examined the expression of G6Pase and PEPCK mRNA, which are involved in gluconeogenesis in the liver. The expression of both G6Pase and PEPCK mRNA was significantly decreased in the liver of SA-treated* db/db* mice compared with PBS-treated mice at 8 weeks (Figures 4(a) and 4(b)). To investigate gluconeogenic fluxes* in vivo*, C57BL/6 mice were orally intubated with SA or PBS. After 15 h later from oral intubation, mice were injected i.p. with sodium pyruvate, and blood glucose levels were measured. SA treatment significantly inhibited blood glucose level at 15 min after pyruvate injection (Figure 4(c)). To investigate the direct effects of SA on hepatic gluconeogenesis* in vitro*, we treated hepatocytes isolated from C57BL/6 mice with SA and then measured glucose production. SA treatment significantly decreased glucose production approximately 70% and 90% at 5 and 10 *μ*M, respectively, as compared with untreated hepatocytes (Figure 4(d)). In addition, the expression of G6Pase and PEPCK mRNA was significantly decreased in SA-treated hepatocytes (Figures 4(e) and 4(f)). The promoter activity of G6Pase and PEPCK is regulated by the transcription factors HNF-4*α*, HNF-3*β*, and FoxO1 [9, 19]. Small heterodimer partner (SHP) has been shown to downregulate G6Pase and PEPCK via the repression of HNF-4*α*, HNF-3*β*, and FoxO1 [20, 21]. Thus, we measured the expression of HNF-4*α* and SHP mRNA in SA-treated hepatocytes. SA treatment significantly decreased the expression of HNF-4*α* mRNA and significantly increased SHP mRNA (Figures 4(g) and 4(h)).

### 3.4. Decreased Expression of Sirt1 mRNA and Protein and Increased Acetylation of FoxO1 in SA-Treated Hepatocytes

**Figure 5:**
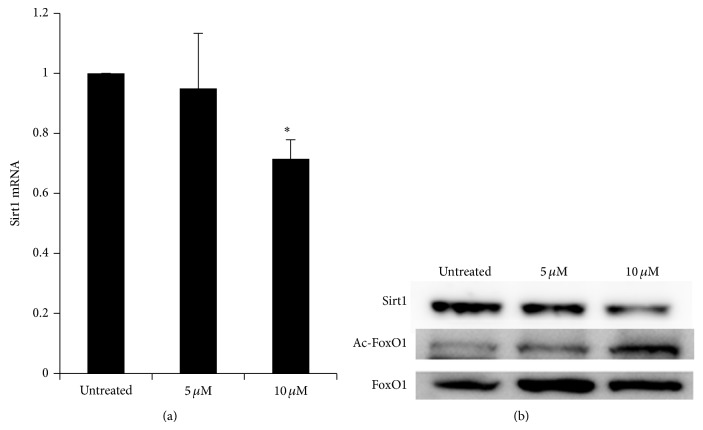
SA inhibited the expression of Sirt1 and increased acetylated FoxO1. (a) Hepatocytes isolated from C57BL/6 mice were incubated with 5 or 10 *μ*M SA for 24 h, and the expression of Sirt1 mRNA was analyzed by RT-qPCR, with values normalized to cyclophilin expression. The fold change was calculated as ratio of the expression in untreated hepatocytes. (b) Hepatocytes were cultured for 48 h with 5 or 10 *μ*M SA. Western blot was performed with antibodies against Sirt1, FoxO1, and acetylated FoxO1 proteins. Data are mean ± SE from three independent experiments. ^*^ *P* < 0.05 compared with untreated hepatocytes.

FoxO1 activity is known to be regulated by phosphorylation and acetylation [22, 23]. Sirt1 increases FoxO1 DNA-binding ability by deacetylating FoxO1 and potentiating its transcription activity [24]. Thus, we determined the expression of Sirt1 and acetylated FoxO1 in SA-treated hepatocytes. The expression of Sirt1 mRNA and protein in SA-treated hepatocytes was significantly decreased compared with untreated control hepatocytes (Figures 5(a) and 5(b)). In addition, acetylated FoxO1 was increased in SA-treated hepatocytes compared with untreated cells (Figure 5(b)).

### 3.5. Decreased Energy Expenditure in SA-Treated* db/db* Mice

**Figure 6:**
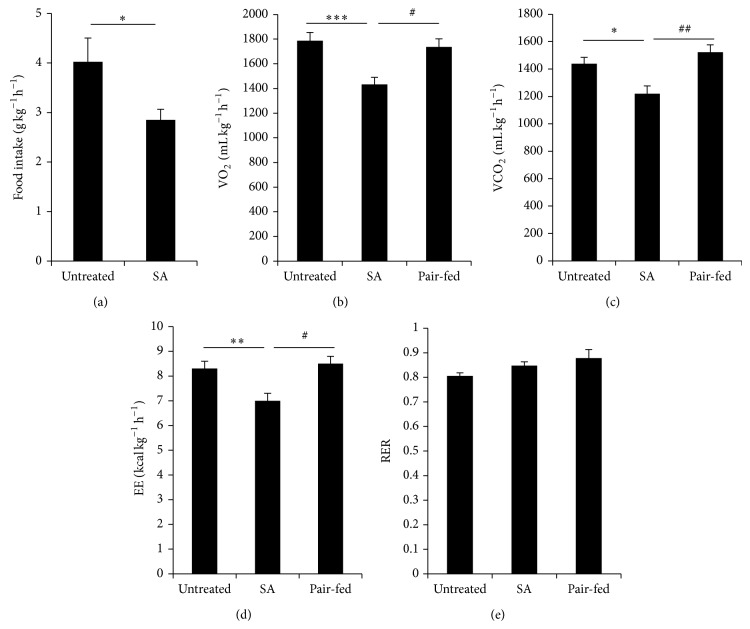
Maintenance of a low level of energy expenditure in SA-treated* db/db* mice. Diabetic* db/db* mice were treated with SA. (a) Food intake, (b) oxygen consumption (VO_2_), (c) carbon dioxide output (VCO_2_), (d) energy expenditure (EE), and (e) respiratory exchange ratio (RER) were measured by indirect calorimetry analysis for 72 h at 8 weeks of SA treatment (*n* = 7-8/group). ^*^ *P* < 0.05, ^**^ *P* < 0.01, and ^***^ *P* < 0.005 compared with untreated mice; ^#^ *P* < 0.05, ^##^ *P* < 0.01 compared with the pair-fed group.

SA-treated mice showed a significant increase in body weight in spite of a significant reduction in food intake (Figures 1(d) and 1(e)). To investigate whether the body weight gain is due to metabolic changes induced by SA treatment, we measured food intake, oxygen consumption, carbon dioxide output, energy expenditure, and respiratory exchange ratio by a metabolic monitoring system over 72 h at 8 weeks of SA treatment. Food intake was significantly decreased in SA-treated mice compared with untreated mice (Figure 6(a)). Oxygen consumption (Figure 6(b)), carbon dioxide output (Figure 6(c)), and energy expenditure (Figure 6(d)) were significantly decreased in SA-treated mice compared with untreated or pair-fed groups. The respiratory exchange ratio was not different in SA-treated or pair-fed mice compared with untreated mice (Figure 6(e)).

**Figure 7:**
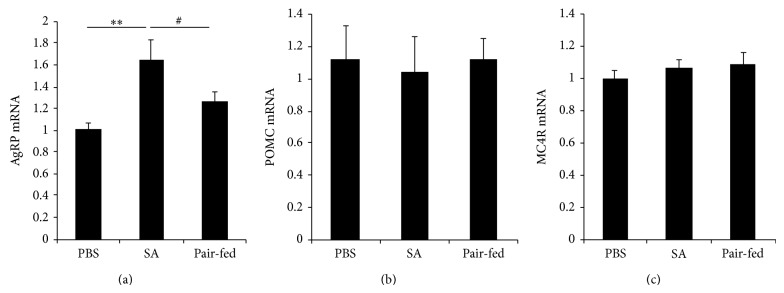
Increase of AgRP gene expression in hypothalamus of SA-treated* db/db* mice. Diabetic* db/db* mice were orally intubated with SA (10 mg kg^−1^ body weight/day) or PBS. Eight weeks later, the hypothalamus was removed, and the expression of (a) AgRP, (b) POMC, and (c) MC4R mRNA was analyzed by RT-qPCR and normalized by cyclophilin expression. The fold change was calculated as a ratio of the expression level in PBS-treated diabetic* db/db* mice. ^*^ *P* < 0.05, ^**^ *P* < 0.01 compared with PBS-treated* db/db* mice (*n* = 5–10/group).

Feeding behavior is regulated by a system with the hypothalamus at the centre, and energy homeostasis is maintained through regulation of food intake and energy expenditure [25, 26]. Therefore, we investigated the expression of hypothalamic molecules known to be involved in appetite regulation, AgRP, proopiomelanocortin (POMC), and a POMC receptor, melanocortin 4 receptor (MC4R), in SA-treated mice. Interestingly, the expression of AgRP mRNA, but not POMC and MC4R mRNA, was significantly increased in SA-treated mice compared with PBS-treated controls and pair-fed group (Figures 7(a)–7(c)).

## 4. Discussion

The liver is an important organ in the regulation of glucose homeostasis in fed and fasting conditions [4]. Excess gluconeogenesis and glycogenolysis in the liver lead to elevated hepatic glucose production, which is a major cause of hyperglycemia in type 2 diabetes [6, 7]. SA has been previously shown to downregulate the expression of the hepatic gluconeogenic genes, G6Pase and PEPCK, in hepatic cell lines, rat hepatocytes, and embryonic chick liver [14, 15, 17]. In this study, we report that SA reduces hepatic gluconeogenesis in diabetic* db/db* mice, an animal model of human type 2 diabetes. SA-treated diabetic* db/db* mice showed decreased blood glucose levels under fed and fasting conditions and improved glucose tolerance. In addition, the expression of gluconeogenesis-related genes was downregulated in SA-treated* db/db* mice liver and mouse hepatocytes.

Gluconeogenesis is a metabolic pathway that generates glucose from noncarbohydrate carbon substrates. This pathway is catalyzed by several enzymes, first and last ones being PEPCK and G6Pase. SA suppressed the expression of PEPCK promoter-driven luciferase constructs in a rat hepatoma H4IIE cell line [14]. SA also repressed forskolin/dexamethasone-stimulated PEPCK and G6Pase gene expression and induced SHP gene expression via AMP-activated protein kinase to inhibit gluconeogenic enzyme gene expression in hepatic cell lines [15]. In our study, G6Pase and PEPCK mRNA levels were significantly reduced in SA-treated diabetic* db/db* mice liver. In addition, HbA1c and fed and fasting glucose levels were significantly decreased in SA-treated diabetic* db/db* mice. These results suggest that SA could improve hyperglycemia in type 2 diabetes through the reduction of gluconeogenesis.

SHP is a member of the nuclear receptor family of intracellular transcription factors [27]. SHP has been shown to inhibit numerous nuclear receptors and transcription factors as transcriptional corepressor [28]. The induction of SHP has been shown to downregulate G6Pase and PEPCK through the repression of HNF-4*α*-, HNF-3*β*-, and FoxO1-mediated transcriptional activity [20, 21]. In our study, the expression of SHP mRNA was significantly increased in SA-treated hepatocytes, and HNF-4*α* mRNA was decreased by SA in a dose-dependent manner in mouse hepatocytes. This suggests that SA-induced increases of SHP work as repressor to inhibit HNF-4*α* expression and subsequently G6Pase and PEPCK production.

FoxO1 is a transcription factor that has an important function in the regulation of gluconeogenesis [29]. Its transcriptional activity is regulated through phosphorylation or acetylation of multiple residues. When FoxO1 is phosphorylated, it is excluded from the nucleus, resulting in decreased transcription of G6Pase and decreased hepatic gluconeogenesis [22]. FoxO1 transcriptional activity is also regulated by acetylation, which inhibits the ability of FoxO1 to interact with the G6Pase promoter by decreasing the stability of the FoxO1-DNA complex [23].

Sirt1 reverses this acetylation process of FoxO1 and has been shown to play critical roles in glucose homeostasis in liver. However, Sirt1 has been shown to confer both positive and negative effects on hepatic gluconeogenesis. The transcriptional activity of FoxO1 is increased by Sirt1 through deacetylation [24]; on the other hand, Sirt1 suppresses the ability of HNF-4*α* to downregulate gluconeogenic gene expression. In our study, the expressions of Sirt1 mRNA and protein levels were significantly decreased depending on SA concentration. It was reported that SA induces NF-*κ*B activation [30], which enhances the expression of miR-34a, a tumor suppressor gene [31]. And SA treatment increases miR-34a expression in TK-6 cells [32], which downregulates the expression of Sirt1 [33]. Thus, the decrease of Sirt1 mRNA by SA in our study may be mediated by the NF-*κ*B/miR-34a pathway. Taken together, all these results suggest that SA decreases Sirt1, thus reducing the deacetylation of FoxO1 and reducing its transcriptional activity, which contributes to the reduced expression of hepatic gluconeogenic genes.

Interestingly, SA-treated* db/db* mice showed reduced food intake over the 8 weeks of the experiment; however, SA-treated mice gained more body weight compared with PBS-treated and pair-fed mice. In addition, SA-treated mice showed decreased consumption of oxygen and production of carbon dioxide, energy expenditure, and locomotor activity compared with pair-fed mice. In the brain, energy balance is regulated by neural and hormonal integrated signals. The hypothalamus is a primary center in which feeding behavior and energy metabolism are regulated [34, 35]. AgRP is a neuropeptide produced in the hypothalamus by the AgRP/neuropeptide Y (NPY) neuron. AgRP increases appetite and decreases metabolism and energy expenditure [36–38]. AgRP treatment for 7 days' intracerebroventricular administration in rat decreased oxygen consumption and energy expenditure [39]. In our study, the expression of AgRP mRNA was significantly increased in SA-treated mice compared with PBS-treated mice, whereas the pair-fed mice were not different from the PBS group. These results suggest that SA decreases energy expenditure and increases weight gain via the induction of AgRP expression in hypothalamus. It is known that FoxO1 regulates AgRP transcription in the hypothalamus, and decreased FoxO1 activity is associated with decreased AgRP expression [40]. However, we found that mRNA expression of AgRP was increased although FoxO1 transcriptional activity was decreased by SA treatment. The expression of AgRP may be regulated by several molecules as well as FoxO1 and further studies are needed for the detailed molecular mechanisms.

It is paradoxical that food intake decreased in SA-treated* db/db* mice in spite of an increase in AgRP, which might be expected to stimulate appetite. In addition, the appetite-stimulating effects of AgRP are inhibited by leptin [41], but* db/db* mice are deficient for the leptin receptor gene [42]. Therefore, the increase of AgRP expression may not affect the appetite in SA-treated* db/db* mice.

In conclusion, our studies show that SA improves glucose metabolism in a type 2 diabetic mouse model. SA directly inhibits hepatic glucose production via regulation of gluconeogenesis-related genes and protein expression, such as PEPCK, G6Pase, SHP, Sirt1, and acetylated FoxO1. In addition, SA increases body weight via decreasing energy expenditure due to induction of AgRP gene expression in hypothalamus. This may benefit end-state lean diabetes patients and increase their longevity.

## Supplementary Material

Supplemental Figure 1: The concentration of ALT and AST in blood of SA-treated db/db mice.
Supplemental Figure 2: Proliferation induced in SA-treated mouse hepatocytes.
Supplemental Figure 3: Glucose-stimulated insulin secretion test in SA-treated db/db mice.
