# Supplementary material for: Sodium Meta-Arsenite Ameliorates Hyperglycemia in Obese Diabetic db/db Mice by Inhibition of Hepatic Gluconeogenesis
Source: J Diabetes Res. 2014 Dec 24;2014:961732. doi: 10.1155/2014/961732 (PMC4290036; doi:10.1155/2014/961732)
Supplement: Supplementary file 1 — Supplemental Figure 1: The concentration of ALT and AST in blood of SA-treated db/db mice. Supplemental Figure 2: Proliferation induced in SA-treated mouse hepatocytes. Supplemental Figure 3: Glucose-stimulated insulin secretion test in SA-treated db/db mice. [file 961732.f1.pdf]

Supplement Fig. 1

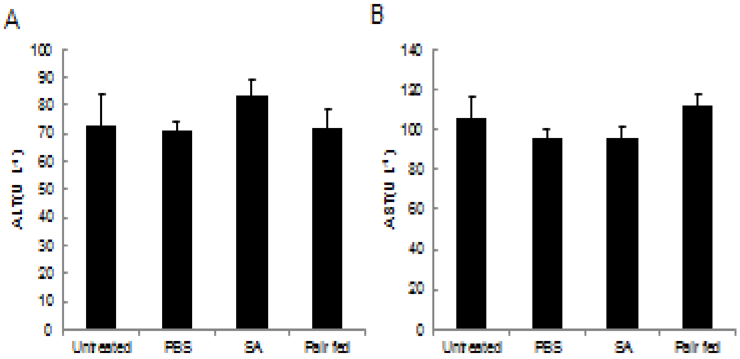

Supplemental Fig. 1. The concentration of ALT and AST in blood of SA-treated *db/db* mice. Diabetic *db/db* mice were orally intubated with SA (10 mg kg^{-1} body weight/day) or PBS. At 8 weeks of SA treatment, the concentration of (A) ALT and (B) AST in the blood was measured (n=4-7 per group).

Supplement Fig. 2

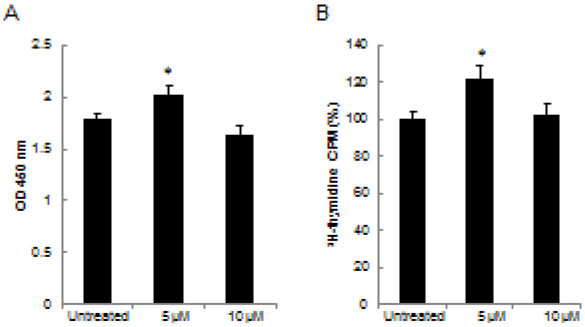

Supplemental Fig. 2. Proliferation induced in SA-treated mouse hepatocytes. (A) Hepatocytes isolated from C57BL/6 mice were incubated with 5 or 10 μM SA for 24 h, and cell toxicity was analyzed by CCK8 assay. (B) Hepatocytes were cultured for 24 h with 5 or 10 μM SA, and cell proliferation was measured by ^{3}H-thymidine incorporation assay. Data are mean ± SE from three independent experiments. * $P<0.05$ compared with untreated hepatocytes.

Supplement Fig. 3

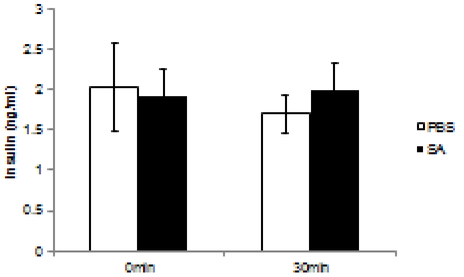

Supplemental Fig. 3. Glucose-stimulated insulin secretion test. After 8 weeks of SA treatment, *db/db* mice were not fed for 14 h, and then a glucose solution (2 g kg^{-1} body weight) was injected intraperitoneally. Blood glucose levels were measured at 0 and 30 min after glucose injection. The concentration of serum insulin was measured by an ultrasensitive mouse insulin enzyme immunosorbent assay kit (ALPCO, Windham, NH). Data are mean ± SE (*n*=5 per group).
